# Targeting mTOR in HIV-Negative Classic Kaposi's Sarcoma

**DOI:** 10.1155/2008/825093

**Published:** 2008-05-15

**Authors:** Ofer Merimsky, Irina Jiveliouk, Ronit Sagi-Eisenberg

**Affiliations:** ^1^Unit of Bone and Soft Tissue Oncology, Division of Oncology, Tel-Aviv Sourasky Medical Center, 6 Weitzman Street, Tel-Aviv 64239, Israel; ^2^Sackler School of Medicine, Tel-Aviv University, Tel-Aviv 64239, Israel; ^3^Department of Cell and Developmental Biology, Sackler School of Medicine, Tel Aviv University, Tel Aviv 69978, Israel

## Abstract

A 66-year old female with HIV-negative classic Kaposi's sarcoma responded to mTOR targeting by rapamycin. The response was well documented by PET-CT. This case provides supporting evidence that the mTOR pathway may be important in the tumorigenesis of KS and that rapamycin may have activity in this disease.

## 1. INTRODUCTION

Kaposi's sarcoma (KS) is an indolent vascularized tumor that has been subdivided into several variants including classic KS, African endemic KS, iatrogenic (post transplantation) KS, and epidemic, acquired immunodeficiency syndrome-associated (AIDS-associated) KS. It behaves more aggressively in HIV-related or in post transplant patients, but its classic form may be more indolent although disfiguring does not commonly result in early mortality. Clinically, KS forms dark blue or purplish macular lesions or spindle-shaped nodular lesions that occur in the skin, lymphoid, respiratory, and gastrointestinal tissues. Histological picture shows networks of spindle-shaped cells and vascular spaces surrounded by an endothelial cell layer. Therapy for localized disease is surgery or radiation therapy, while widespread disease may be treated by systemic chemotherapy [1]. A novel approach for the treatment of iatrogenic KS is mTOR targeting by rapamycin. Rapamycin is currently available as an antirejection agent and may be also used as antitumoral therapy [2, 3]. In this paper, we report a case of HIV-negative classic KS, which responded to mTOR targeting.

## 2. CLINICAL CASE

**Figure 1:**
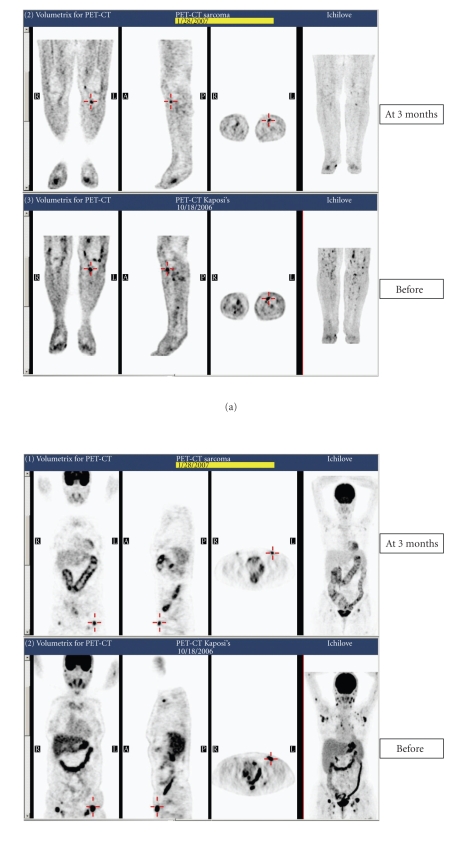
PET-CT at initiation and at completion of almost 4-month rapamycin therapy.

A 66-year old otherwise healthy Caucasian female patient presented with metastatic HIV-negative Kaposi's sarcoma (KS), diffusely involving the skin and soft tissues of the plantar surface, legs, and thighs. During a prolonged interval of palliative radiation therapy and various ineffective systemic chemotherapeutic regimens that included vinblastine, etoposide, doxorubicin, and alpha-interferon. The disease progressed and spread to the bowel and to the soft tissues of the left arm and to the inguinal, mediastinal, axillary, and cervical nodes. Clinically, the patient had a Karnofsky's performance status of 80–70%, and complained of weakness, and pain in her lower limbs. Radiologically, the disease was well documented by PET-CT performed on October 18, 2006 (Figure 1). After obtaining a local IRB approval and signing an informed consent, oral rapamycin was started according to the following schedule: 1 mg daily in the 1st week, 1 mg twice daily in the 2nd week, 1 mg three times daily in the 3rd week, and then 1 week off. The next 28-day cycles included oral rapamycin 1 mg three times daily for 3 out of 4 weeks. No serum level of rapamycin was obtained as in cases of organ transplantation. After a 4-week uneventful induction, she moved to 3 mg daily rapamycin. Side effects included anemia (Hb 9.8 gr/dl), weakness, and mild stomatitis. On January 2007, about 3 months after treatment initiation, the soft tissue lesions partially regressed, and the patient mentioned itching in the legs and trunk. PET-CT, performed at the end of January 2007, demonstrated significant decrease in FDG uptake in almost all involved sites (Figure 1). The patient is still on treatment for almost 12 months now.

## 3. DISCUSSION

Our case points to two important observations. First is the role of mTOR as a therapeutic target in HIV-negative classic KS. We observed that rapamycin monotherapy was more active and better tolerated than systemic, conventional chemotherapy in this patient. The second is monitoring of the objective response by PET-CT imaging. The clinical improvement correlated with metabolic shutdown of glucose metabolism in the sarcoma nodules, as documented by decrease in FDG uptake. PET-CT as a tool for staging and monitoring of therapy has been suggested for vascular tumors [4].

Rapidly accumulating data implicate rapamycin, a natural product produced by Streptomyces hygroscopicus as a potential anticancer agent. While originally identified 20 years ago during antibiotic screening and found to display remarkable antifungal activity, rapamycin was subsequently recognized to possess highly potent immunosuppressive properties [5] and has since been used as the drug of choice in organ transplantation. More recently, the growth inhibitory effects of rapamycin have been recognized alongside the elucidation of the molecular basis of its function. The ultimate cellular target of rapamycin has been identified as a signaling kinase named “mTOR” (mammalian target of rapamycin), a member of the phosphatidylinositol-3-kinase (PI3K)-related kinases (PIKKs). The PIKK family members, also including the ATM kinase, are involved in the control of essential cell functions, including cell-cycle progression, cell-cycle check points, DNA repair, and DNA recombination [6]. Specifically, mTOR is a downstream component in the PI3K/Akt (protein kinase B) pathway, which participates in the activation of the p70S6 kinase (p70S6k). In response to mitogenic stimuli, PI3K is activated resulting in a rise in the cellular concentration of PIP_3_. This in turn leads to activation of Akt followed by activation of mTOR. MTOR then phosphorylates and activates p70S6k, the kinase which phosphorylates the ribosomal protein S6 thereby promoting the recruitment of the 40S subunit into actively translating polysomes [7]. Following mitogenic stimulation and activation of mTOR, the latter also phosphorylates the 4E-binding protein-1 (4E-BP1). The latter binds tightly to eIF-4E thereby preventing initiation of translation. However, upon its mTOR-mediated phosphorylation, 4E-BP1 dissociates thereby allowing eIF-4E to bind to the eIF-4F complex and translation increases [8, 9]. Therefore, mTOR activation results in the transduction signaling events that ultimately activate cyclin-dependent kinases (CDKs), increase cellular levels of cyclins such as cyclin D1, and promote retinoblastoma (Rb) protein phosphorylation. As such, mTOR plays a central role in the control of cell proliferation, cell survival, and adhesion-independent survival and migration [10, 11].

Rapamycin binds to its intracellular receptor protein FKBP12 [12], forming a complex, which then binds to and inhibits the function of mTOR [12]. Through mTOR inhibition, rapamycin causes cell cycle arrest in the G1 phase, prevents CDK activation, inhibits Rb protein phosphorylation, and accelerates the turnover of cyclin D1, leading to a deficiency of active CDK4/cyclin D1 complexes. These events then contribute to the prominent inhibitory effects of rapamycin at the G1/S boundary of the cell cycle [13]. Notably, rapamycin also displays antiangiogenic activities linked to a decrease in production of vascular endothelial growth factor (VEGF) thereby markedly inhibiting response of vascular endothelial cells to stimulation by VEGF [14].

The antiproliferative activity of rapamycin was first evaluated in variety of murine tumor cell lines and tumor model systems. In those experiments, rapamycin was found to exhibit impressive antitumor activity inhibiting the growth of lymphoma cell lines, small cell lung cancer cell lines, rhabdomyosarcoma cell lines, pancreatic cancer cell lines and more (reviewed in [15]). Moreover, rapamycin augmented cisplatin-induced apoptosis [16] and conferred radiation sensitivity to otherwise resistant tumor cell lines [17]. Taken together, these results suggest that rapamycin has both intrinsic antiproliferative activity against a broad range of cancers and the ability to synergize and enhance efficacy of cytotoxic agents [18]. Indeed, clinical trials of CCI-779 and Rad 001, structurally related analogs of rapamycin with increased water solubility, are currently ongoing in recurrent malignant glioma patients, in renal cell carcinoma, metastatic pancreatic cancer, endometrial cancer, and more.

The role of mTOR signaling pathway in pathogenesis of Kaposi's sarcoma has been elucidated [19]. A human herpes virus 8 (HHV8) or Kaposi's sarcoma associated herpes virus (KSHV) has been suggested as the etiological agent for this tumor [20]. A single lytic gene, the KSHV G protein-coupled receptor (vGPCR), a member of the family of CXC chemokine GPCRs which exhibits ligand-independent activity, has been shown to be responsible for the initiation of KS [21]. Infection with KSHV is necessary and central to the development of KS. KSHV infects KS spindle and endothelial cells in KS lesions, seen eventually in all four forms of KS (classic (Mediterranean), AIDS-related, endemic (African) and iatrogenic) [22].

The discovery that the development of KS may be dependent on vGPCR dysregulation suggests that the development of new mechanism-based therapeutic modalities specifically targeting this viral protein (or its downstream targets) may prove to be an effective treatment strategy [19]. Several intracellular proteins, kinases, and transcription factors have been shown to be involved in vGPCR-expressing cells [19]. It is believed that KSHV is the putative gene encoding the vGPCR protein that is responsible for the development of KS, via dysregulation mechanisms in certain circumstances [19]. It has been suggested that anti-KS therapy might target the viral protein or the downstream molecules involved in the KS pathogenesis [19]. The vGPCR leads to activation of the PI3K/Akt pathway [19]. Activation of Akt was found to be involved in the protection of endothelial cells from apoptosis, especially in KSVV-infected cells [19, 23]. AkT plays an essential role in the activation of mTOR via inactivation of tuberous sclerosis complex (TSC) [24, 25]. The TSC/mTOR pathway may play a critical role in the development of tumors dependent on Akt activation, and the pathway may be regulated by vGPCR via inactivation of TSC, and consequent mTOR activation [19]. Other mechanisms for KS genesis have also been suggested [19].

In conclusion, this case provides supporting evidence that the mTOR pathway may be important in the tumorigenesis of KS and that rapamycin may have activity in this disease. A phase II study for assessing the exact role of mTOR inhibitors in this disease is warranted.
